# Ammonia-Oxidizing Bacteria Rather than Ammonia-Oxidizing Archaea were Widely Distributed in Animal Manure Composts from Field-Scale Facilities

**DOI:** 10.1264/jsme2.ME12053

**Published:** 2012-09-05

**Authors:** Nozomi Yamamoto, Ryu Oishi, Yoshihisa Suyama, Chika Tada, Yutaka Nakai

**Affiliations:** 1 Laboratory of Sustainable Environmental Biology, Graduate School of Agricultural Science, Tohoku University, 232–3 Yomogida, Naruko-onsen, Osaki, Miyagi 989–6711, Japan; 2 Laboratory of Forest Ecology, Graduate School of Agricultural Science, Tohoku University, 232–3 Yomogida, Naruko-onsen, Osaki, Miyagi 989–6711, Japan

**Keywords:** ammonia-oxidizing archaea (AOA), ammonia-oxidizing bacteria (AOB), animal manure compost, PCR-DGGE, real-time PCR

## Abstract

The distribution of ammonia-oxidizing archaea (AOA) and bacteria (AOB) in cattle, swine, and chicken manure compost was analyzed. PCR-denaturing gradient gel electrophoresis (DGGE) showed that a *Candidatus* Nitrososphaera gargensis-like sequence dominated in cattle manure compost, while few AOA were detected in other composts. In the case of AOB, *Nitrosomonas*-like sequences were detected with higher diversity in cattle and swine manure composts. The relative abundance of ammonia oxidizers by real-time PCR revealed that more AOB was present in compost except in one swine manure compost. Our results indicated that AOB rather than AOA are widely distributed in animal manure compost.

Nitrification with ammonia oxidation occurs naturally during treatment of animal manure via composting (29). It is viewed as a key process in N dynamics, because nitrogen loss impacts compost quality as well as environmental health and pollution (*e.g.*, NH_3_ emission and nitrate leaching) through nitrification (1, 18). Ammonia oxidizers are key players because ammonia oxidation is a rate-limiting process in autotrophic nitrification (11). Until recently, it was believed that only ammonia-oxidizing bacteria (AOB) oxidize ammonia to nitrite in the composting process. In a previous study, both *Nitrosomonas* spp. and *Nitrosospira* spp. clusters were present throughout the composting process (6, 7, 10, 16). But in recent years, archaeal organisms, called ammonia-oxidizing archaea (AOA), have been discovered in soil and marine environments and are significant contributors to ammonia oxidation (8, 28). A positive relationship was also observed between the potential ammonia oxidizing (PAO) rate and archaeal *amoA* gene abundance during thermophilic and cooling stages of composting from agricultural waste (35). As for cattle manure compost, one study detected the archaeal *amoA* gene and related 16S rRNA gene with low diversity (31, 32). Our group also revealed that an archaeon related to *Candidatus* Nitrososphaera gargensis was dominant in liquid cultures seeded with cattle manure compost (20); however, there is no information about AOA community in other animal manure compost. To clearly understand the nitrification process in compost ecosystems, we researched ammonia oxidizers in various animal composts.

Compost samples were collected from November 2009 to November 2010 from ten different facilities (Table 1). Compost samples were obtained approximately 30 cm from the surface of the source material. Manure samples were obtained from the surface of the material. Water content was determined by wet weight minus dry weight after placing in a drying oven at 105°C overnight. PH was measured using compost suspension with a pH meter (WM-22EP; DKK-TOA, Tokyo, Japan). To estimate the concentration of inorganic nitrogen ions, materials were made into a compost suspension by the addition of deionized water in a compost : water ratio of 1:10. The suspension was then filtered through the 0.45 μm cellulose acetate filter (Advantec Toyo, Tokyo, Japan) to provide for ICS-1000 and ICS-2000 ion chromatography systems (Thermo Fisher Scientific, Waltham, MA, USA). The carbon:nitrogen (C:N) ratio was calculated using triplicate freeze-dried samples with an N and C determination unit (Sumigraph NC-80S; Sumika Chemical Analysis Service, Osaka, Japan). Chemical and physical properties of the samples are listed in Table 2. Total DNA was extracted from 0.05 g freeze-dried samples using the Favorgen DNA Isolation kit (Favorgen Biotech Corporation, Taiwan) in duplicate. After purification by ethanol precipitation with PelletPaint NF Co-Precipitant (Merck KGaA, Darmstadt, Germany), the bacterial *amoA* gene and archaeal *amoA* gene were amplified with the primer set amoA1F/amoA2R-I (5) and AOA23F/AOA616R (26), respectively. A GC clump was attached to the forward primer amoA1F (5). PCR and DGGE conditions were as described by Yamamoto *et al.* (32), with some modification. *Taq* DNA polymerase (TaKaRa, Shiga, Japan) was used to amplify the bacterial *amoA* gene. Gel staining, band excision, and sequencing of DNA bands were performed in accordance with Yamamoto *et al.* (31). Phylogenetic trees for the archaeal and bacterial *amoA* genes were constructed using Molecular Evolutionary Genetics Analysis (MEGA) software ver. 4.0.1 (24). Unrooted phylogenetic trees were generated using the neighbor-joining method and bootstrap tests were performed with 1,000 replicates.

AOB and AOA *amoA* gene copy numbers were determined using real-time PCR for samples that confirmed the presence of each organism by PCR amplification. PCR amplification was conducted with SYBR Premix Ex *Taq* II (TaKaRa) using the primer sets amoA1F/amoA2R (23) and AOA23F/AOA616R (26), respectively. As standards for real-time PCR, plasmid DNA, including the bacterial *amoA* gene from compost (for AOB) or the archaeal *amoA* gene from enrichment culture (for AOA [20]) was used. PCR conditions and statistical tests were performed as described by Yamamoto *et al.* (32).

One DGGE band, indicated as group NG in Fig. 1, was ubiquitous in the profiles of cattle manure compost, and was closely related (97–100% sequence identity) to a sequence previously detected from cattle manure compost (31). This sequence was grouped into *Candidatus* Nitrososphaera gargensis, which are enriched from 46°C hot springs (4) and can resist high temperatures. The DGGE profile from facility A generated several bands, indicating that the AOA community was relatively diverse. In samples from other facilities, only one to four bands were detected. In the case of swine manure compost, samples from one of three facilities resulted in detectable PCR amplification. The presence of AOA in swine manure compost was thought to be rare because another study reported that the archaeal community consisted of mainly methanogen and uncultured Crenarchaeota (12). Another sequence, which was identical to the sequence found in cattle manure compost, was detected in samples from facility H. Because the finished compost could have a diverse microbial community (25), the archaeal *amoA* sequence detected in facility H may have been the result of a two-fold amount of finished compost being mixed with fresh manure as the starting material for composting.

No archaeal *amoA* sequences were detected in any fresh manure samples, in agreement with a previous study in which PCR failed to amplify archaeal *amoA* gene sequences from cattle manure (31). In addition, the archaeal *amoA* gene was not amplified using mixed samples of cattle and swine manure. Similarly, PCR also failed to amplify sequences from samples of chicken manure compost. Further research is needed to confirm which condition is critical for the survival of ammonia oxidizers in animal manure compost.

Our results indicate that AOA might be a minor part of the microbial community of animal manure compost, with the exception of cattle manure, because few archaeal *amoA* sequences were observed in swine and chicken manure composts. Nonetheless, it seems that specific AOA species can survive in cattle manure compost under harsh conditions and environmental factors. One possible factor for the presence of AOA is relatively low ammonium concentration, as a previous study reported (2, 8, 17). In natural environments, such as seawater and soil, the ammonium concentration may be the key determinant of AOA niche (3). There is little information on AOA that live in environments with high ammonium concentrations, such as activated sludge, and therefore few AOA have been identified (30, 34). Notably, *Ca.* Nitrososphaera gargensis-like sequences were not detected in composting materials with >3.1 mg N kg^−1^ (fresh weight), which is equivalent to 221 mM ammonium. Related AOA is active at 0.14 and 0.79 mM ammonium and is partially inhibited by a concentration of 3.08 mM (4). Our previous study found that archaeal *amoA* copy numbers increase significantly at 46°C and 10 mM ammonium after 2-week incubation of cattle manure compost inoculated into liquid culture (20). *Nitrososhaera viennensis*, which was originally isolated from garden soil, showed a similar physiological property of growing in ammonium concentrations of up to 15 mM (27); therefore, *Nitrososphaera* spp. could survive under relatively higher ammonium concentrations. Another possible factor was the changes in temperature during the composting process. It was unsurprising that detected *amoA* sequences were derived from moderately thermophilic AOAs since *Ca.* Nitrososphaera gargensis, to which most sequences were related, was enriched at 46°C (4). Some AOA, such as those found in compost, are resistant to high temperatures (22, 33); however, we did not detect *Ca.* Nitrososphaera gargensis-like sequences in samples from Facility J, despite their low levels of ammonium (1.177 and 0.407 g N^−1^DM).

Park *et al.* (21) investigated various activated sludge samples from nine wastewater plants and found that both *amoA* genes were detected at four plants, whereas some plant samples had only one gene. The authors of that study speculated that low dissolved oxygen (DO) levels and long retention times contributed to the growth of AOA. Like activated sludge, other factors, such as oxygen concentration, may have affected which AOA species dominated the composting material.

As shown in the phylogenetic tree, the bacterial *amoA* sequence showed greater diversity in fresh manure than in compost in the high-temperature or mature stage (Fig. 2). Low diversity of the AOB *amoA* sequence was previously reported in a study of the cattle manure composting process, indicating that the composting process was a harsh environment for AOB species (32). Maeda *et al.* (16) reported a large number of bacterial *amoA* sequences in the surface of compost piles, depending on the accumulation of nitrite or nitrate. Our results are consistent with this, and furthermore confirm that particular AOB species survive and actively grow at high temperatures in each composting process. By sequencing analysis, almost all sequences were defined as *Nitrosomonas*-like *amoA* gene sequences. This finding is consistent with previous reports that *Nitrosomonas europaea-*like and/or *Nitrosomoas halophila*-like *amoA* genes dominated the cattle manure composting process (16, 32). *Nitrosospira*-like *amoA* sequences were found only in the final product from facility C. A strong band occurred in all samples and had an identical sequence to that derived from cattle manure compost (31). In addition, another band was highly similar (99%) to that of the same cattle manure compost sample. The diversity of the AOB community was low in the samples derived from swine manure compost, as only 2–3 bands appeared in each sample; however, AOB composition differed among samples. In facility F, two sequences appeared consistently and were related to sequences isolated from activated sludge and cattle manure compost, respectively. Sequences detected in raw manure disappeared and new sequences appeared in other samples from facility H, suggesting that the AOB community structure changed during the composting process. These findings were caused by differences in the chemical parameters, such as ammonium concentration. It was suggested that a low ammonia level might be the key factor dominating the *N. oligotropha* cluster in most wastewaster treatment systems studied by Limpiyakorn *et al.* (15). They also described that factors such as ammonia affinity, oxygen affinity, sensitivity to salt and/or nitrite may affect sequence types. Identifying critical factors for AOB sequence diversity requires further study. When chicken manure compost was analyzed by PCR, no amplification was observed for five of six samples. Only the sample from the high-temperature phase of composting from facility J had two DGGE bands, but PCR amplification was not successfully repeated in duplicate. This indicated that few AOB were present in chicken manure compost. A previous study also showed no DNA amplification from chicken manure by PCR for the *amoA* gene (10).

Compost material contained bacterial *amoA* sequences at high temperature (>50°C), although AOB are grown under mesophilic conditions (9). In the composting process, *Nitrosomonas europaea*-like sequences have been detected under thermophilic conditions (7, 16). Our findings strongly supported the hypothesis that uncultured thermophilic AOB is present in the high-temperature stage of composting. This is very rare, because only one previous AOB enrichment culture, derived from a hot spring, grew at temperatures of 27–55°C (13).

In the analysis of AOB, minor variations between duplicate samples occurred in samples from facilities A, C, and J, even though the samples were freeze-dried and homogenized. This may point to variability in the DNA extraction step, because small sample aliquots were used. Nicol *et al.* (19) revealed that DGGE profiles generated from triplicate 0.1 g samples showed great heterogeneity, whereas triplicate 10 g samples had similar patterns. In this study, the DGGE band pattern was slightly different between duplicate DNA samples in some materials due to the low abundance of the targeted organism rather than heterogeneity of the material, which was indicated in real-time PCR (Fig. 3). However, it is believed that compost materials have large spatial variation as the distribution of the AOA community was demonstrated in animal manure composts. Mixing core samples collected from different locations enables analysis of the community structure dynamics of AOA during the composting process.

In cattle manure compost from facilities A and C, the abundance of archaeal *amoA* genes (4.58 and 6.57×10^6^ copies g^−1^ DW, respectively) was clearly lower than that of bacterial genes (8.77×10^7^ and 1.03×10^8^ copies g^−1^ DW, respectively) (Fig. 3). Both *amoA* gene copy numbers were below the limits of detection (≤10^5^ copies g^−1^ DW) in samples from facility B. These findings are in contrast to our previous study in which AOA may have been more abundant than AOB during the cooling and maturation stages of composting (32). Real-time PCR confirmed that archaeal *amoA* gene copy numbers (2.55×10^7^ g^−1^ DW) were greater than bacterial gene copy numbers (2.42×10^6^ g^−1^ DW) in the end product at facility H. The AOA:AOB ratios varied from 0.06 to 10.54. Differences in the AOA:AOB ratio were also observed in the tested soil samples, ranging from 1.5 to >230 (14). This variation might have been caused by the operating conditions of composting, such as the addition of finished compost; however, it is unclear which critical factor influences the ratio of AOA to AOB in compost. In this study, it was indicated that AOB rather than AOA contributed to nitrification in animal manure composting.

In summary, the distribution and abundance of ammonia oxidizers were analyzed in cattle, swine, and chicken manure composting systems. Specific AOA were detected in cattle manure compost samples and in one swine manure sample tested; however, the proportion of AOA to AOB gene numbers varied, suggested that AOB rather than AOA was distributed in animal manure compost. One possible environmental factor that helps define the ecological niche for ammonia oxidizers may be ammonia concentration, although other factors may also play a role. Based on the present study, it is worth investigating the activity and contribution of AOB to ammonia oxidation during animal manure composting processes; however, it is not impossible that novel AOA species are present in compost, because the primer set used in this study possibly did not amplify all types of the archaeal *amoA* gene. To measure the contribution of each ammonia oxidizer to the ammonia oxidation process during composting, further studies are needed to evaluate ammonia oxidation activity under changing chemical parameters, such as ammonia concentration and water content, while using the same compost material. In addition, it is also necessary to use multiple primer sets for PCR amplification of the targeting gene.

## Supplementary Material



## Figures and Tables

**Fig. 1:**
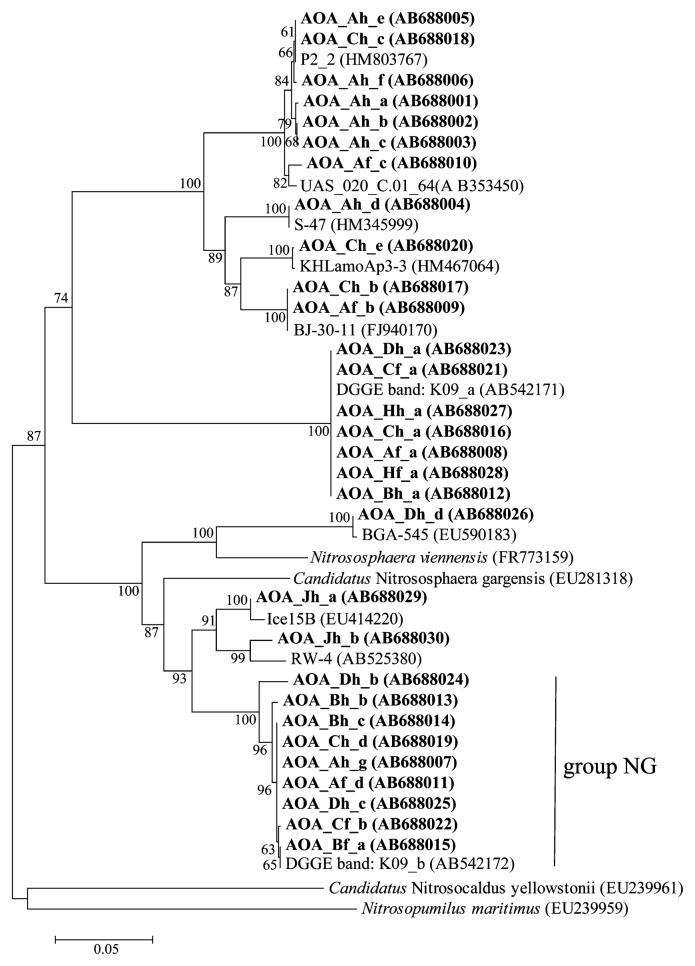
Phylogenetic tree of the archaeal *amoA* sequences obtained from composting materials. Boot-strap values (>60%) are indicated at the branch points. All sequences obtained from this study are given in bold. Capital and small letters after ‘AOA_’ indicate the sampling facility and sampling point, respectively. Samples were obtained from each facility from fresh manure (m), the high-temperature stage (h), and the end of composting (f). The last letter indicates the band name. Scale bar represents 5% sequence divergence. Accession numbers are given in parentheses.

**Fig. 2:**
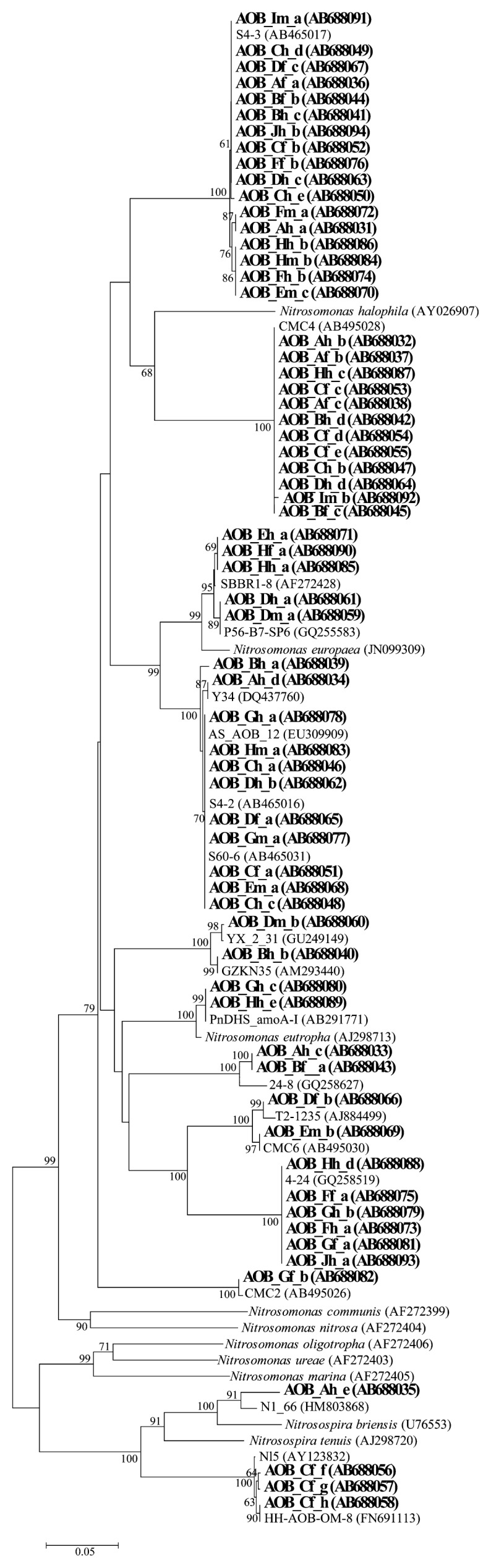
Phylogenetic tree of the bacterial *amoA* sequences obtained from composting materials. Boot-strap values (>60%) are indicated at the branch points. All sequences obtained from this study are given in bold. Capital and small letters after ‘AOB_’ indicate the sampling facility and sampling point, respectively. Samples were obtained from each facility from fresh manure (m), the high-temperature stage (h), and the end of composting (f). The last letter indicates the band name. Scale bar represents 5% sequence divergence. Accession numbers are given in parentheses.

**Fig. 3:**
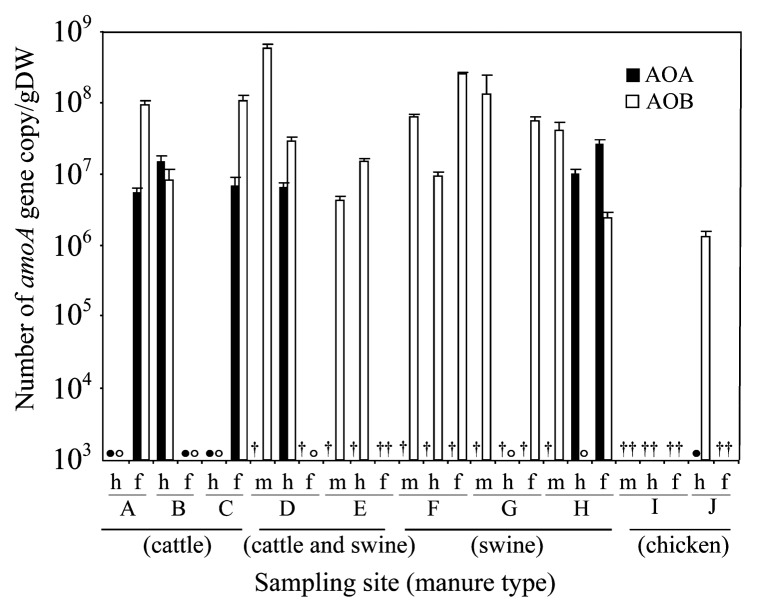
*AmoA* gene copy numbers for AOA and AOB. Error bars indicate standard deviation. Each sample was analyzed in triplicate. Samples with gene copy numbers below the detection limit are represented by closed circles (AOA) and open circles (AOB). Capital letter indicates sampling facility. Samples were obtained from each facility from fresh manure (m), the high-temperature stage (h), and the end of composting (f). Samples with a dagger did not undergo real-time PCR.

**Table 1:** Information of samples analyzed in this study

Facility	Main raw manure	Amendments	Processing conditions					

			1^st^ period			2^nd^ period		

			Type^a^	No. of days	Aeration	Type^a^	No. of days	Aeration
A	Dairy cattle	Rice chaff	open	20	yes	pile	40	no
B	Beef cattle	Rice chaff	open	30	yes	pile	20	no
C	Dairy and beef cattle	Rice chaff	open	20	yes	pile	30	no
D	Cattle and swine	Rice chaff and finished compost	open	20	yes	pile	40	no
E	Cattle and swine	Rice chaff and finished compost	open	10	yes	ND	14	ND
F	Swine	Rice chaff	pile	60	no	—	—	—
G	Swine	Sawdust, finished compost and rice bran	open	25	yes	—	—	—
H	Swine	Finished compost	open	25	yes	—	—	—
I	Chicken	Finished compost	pile	70	yes	—	—	—
J	Chicken	Bark and finished compost	pile	ND	yes	open	ND	yes

* , open type means the type that composting material was turned by turning conveyor belt or rotary device, and pile type means the type that material was turned by shovel loader.

ND, no data; —, not conducted.

**Table 2:** Chemical parameters of samples analyzed in this study

Facility	Composting stage^a^	Chemical parameters							

		Temperature (°C)	Water content (%)	pH	Total N (%DM)	C/N ratio	NH_4_^+^ (gN/kgDW)	NO_2_^−^ (gN/kgDW)	NO_3_^−^ (gN/kgDW)
A	h	52.0	58.53	9.18	1.9	19.3	0.480	ND	0.118
	f	20.7	61.02	9.16	2.2	14.7	0.167	ND	0.591
B	h	70.4	68.59	9.05	1.9	19.5	3.101	ND	0.165
	f	22.4	28.10	7.77	2.6	15.4	0.917	ND	2.509
C	h	70.9	55.53	9.17	2.4	15.0	1.462	ND	0.111
	f	32.5	39.53	8.80	2.4	14.9	0.290	ND	0.421
D	m	NA	75.35	7.04	2.8	16.3	8.200	ND	ND
	h	61.1	59.71	9.53	1.8	20.3	1.877	ND	ND
	f	38.3	30.70	9.66	1.9	18.5	0.745	ND	0.007
E	m	NA	74.93	6.26	3.9	21.8	10.290	ND	ND
	h	72.7	53.70	8.83	2.7	13.4	4.234	ND	0.005
	f	29.0	25.23	9.45	2.5	12.7	1.073	ND	0.167
F	m	NA	69.79	NA	2.2	17.9	7.610	ND	ND
	h	68.7	57.39	NA	1.3	26.1	1.271	0.062	0.053
	f	43.3	45.03	NA	1.4	21.8	0.710	ND	0.054
G	m	NA	77.44	5.97	3.0	14.1	7.300	ND	ND
	h	59.0	42.33	7.97	2.9	13.5	4.439	ND	0.002
	f	35.0	29.86	8.50	3.5	9.9	1.658	ND	1.192
H	m	NA	72.48	8.12	3.8	10.7	3.210	ND	ND
	h	71.5	44.63	8.50	5.3	7.3	5.569	ND	0.006
	f	36.9	24.26	8.78	5.4	7.1	2.582	ND	0.069
I	m	NA	66.90	6.43	4.8	5.7	3.650	ND	0.001
	h	72.0	28.34	8.65	4.8	5.9	2.807	0.032	0.051
	f	35.0	18.04	8.64	3.9	6.5	2.351	ND	0.219
J	h	67.1	54.59	9.86	1.7	19.2	1.177	ND	0.001
	f	51.3	40.04	10.02	1.9	17.2	0.407	ND	0.034

a , m indicates fresh manure, h indicates high temperature stage, and f indicates finished compost.

ND, not detected; NA, not analyzed.
