# Supplementary material for: Ammonia-Oxidizing Bacteria Rather than Ammonia-Oxidizing Archaea were Widely Distributed in Animal Manure Composts from Field-Scale Facilities
Source: Microbes Environ. 2012 Sep 5;27(4):519–24. doi: 10.1264/jsme2.ME12053 (PMC4103565; doi:10.1264/jsme2.ME12053)
Supplement: Supplementary file 1 [file 27_519_s1.pdf]

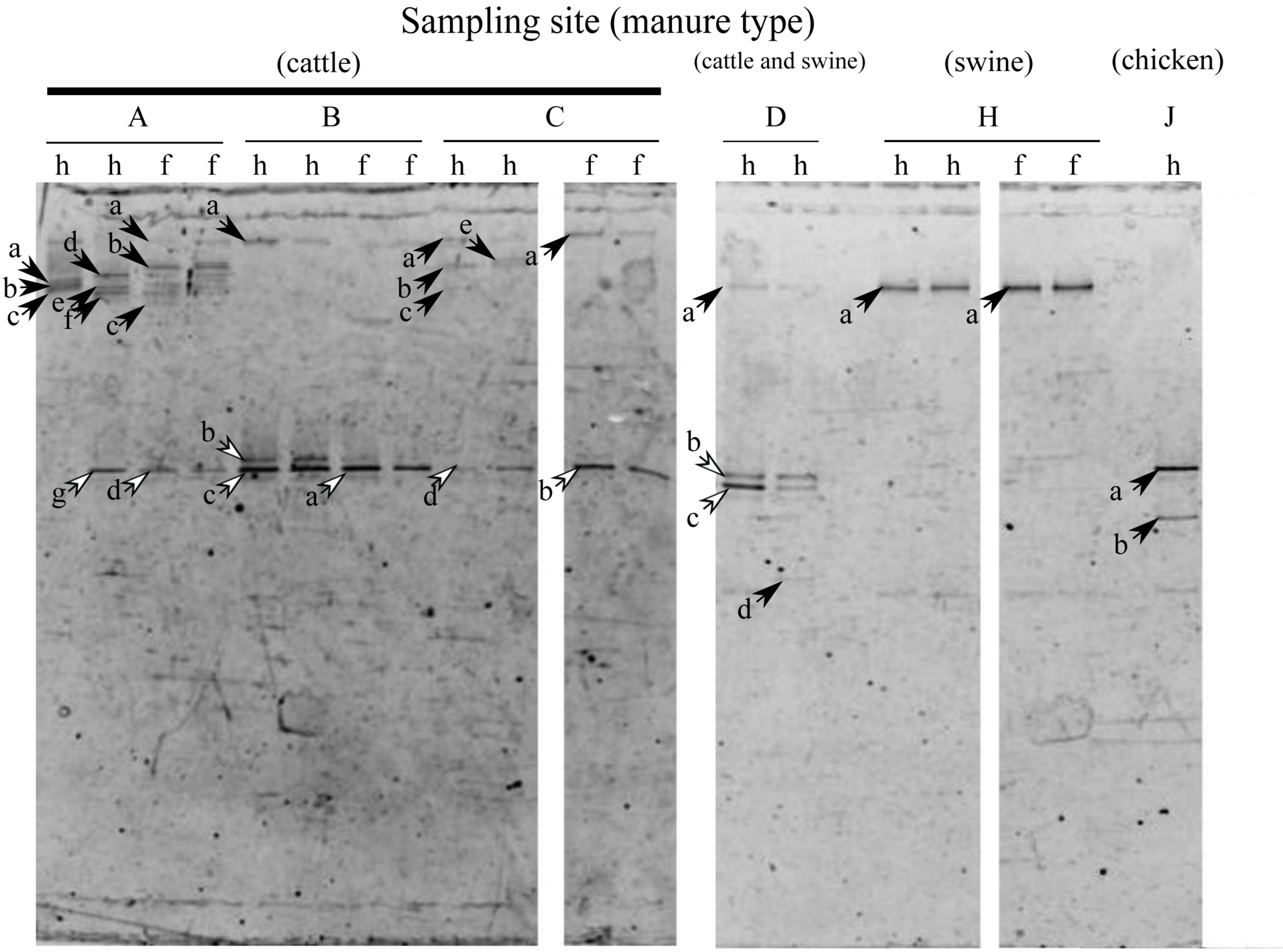

Fig. S1. DGGE profiles of the compost samples for the archaeal *amoA* gene. Black and white arrows indicated bands sequenced. DGGE bands indicated white arrows were identified as the member of group NG.

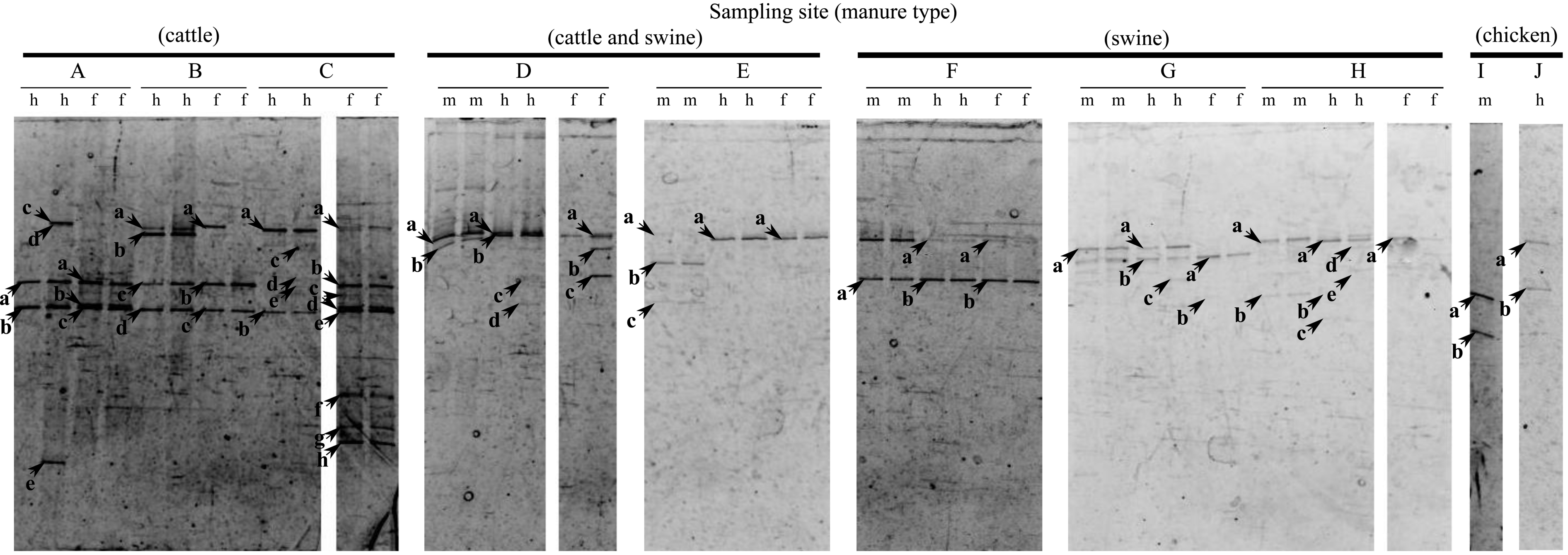

Fig. S2. DGGE profiles of the compost samples for the bacterial *amoA* gene. Black arrows indicated bands sequenced.
